# Age Differences in Perceptions of and Motivations for Voluntary Medical Male Circumcision Among Adolescents in South Africa, Tanzania, and Zimbabwe

**DOI:** 10.1093/cid/cix951

**Published:** 2018-04-03

**Authors:** Eshan U Patel, Michelle R Kaufman, Kim H Dam, Lynn M Van Lith, Karin Hatzold, Arik V Marcell, Webster Mavhu, Catherine Kahabuka, Lusanda Mahlasela, Emmanuel Njeuhmeli, Kim Seifert Ahanda, Getrude Ncube, Gissenge Lija, Collen Bonnecwe, Aaron A R Tobian

**Affiliations:** 1 Department of Pathology, Johns Hopkins University School of Medicine, Baltimore, Maryland; 2 Johns Hopkins Bloomberg School of Public Health, Baltimore, Maryland; 3 Johns Hopkins University Center for Communication Programs, Baltimore, Maryland; 4 Population Services International, Harare, Zimbabwe; 5 Department of Pediatrics, Johns Hopkins University School of Medicine, Baltimore, Maryland; 6 Centre for Sexual Health & HIV/AIDS Research, Harare, Zimbabwe; 7 CSK Research Solutions, Dar es Salaam, Tanzania; 8 Centre for Communication Impact, Pretoria, South Africa; 9 Office of HIV/AIDS, Global Health Bureau, United States Agency for International Development, Washington, District of Columbia; 10 Ministry of Health and Child Care, Harare, Zimbabwe; 11 Ministry of Health, Community Development, Gender, Elderly and Children, Dar es Salaam, Tanzania; 12 National Department of Health, Pretoria, South Africa

**Keywords:** adolescent health, male circumcision, HIV, norms, stigma, motivation

## Abstract

**Background:**

The World Health Organization (WHO) and the Joint United Nations Programme on HIV/AIDS (UNAIDS) have set a Fast-Track goal to achieve 90% coverage of voluntary medical male circumcision (VMMC) among boys and men aged 10–29 years in priority settings by 2021. We aimed to identify age-specific facilitators of VMMC uptake among adolescents.

**Methods:**

Younger (aged 10–14 years; n = 967) and older (aged 15–19 years; n = 559) male adolescents completed structured interviews about perceptions of and motivations for VMMC before receiving VMMC counseling at 14 service provision sites across South Africa, Tanzania, and Zimbabwe. Adjusted prevalence ratios (aPRs) were estimated using multivariable modified Poisson regression models with generalized estimating equations and robust standard errors.

**Results:**

The majority of adolescents reported a strong desire for VMMC. Compared with older adolescents, younger adolescents were less likely to cite protection against human immunodeficiency virus (HIV) or other sexually transmitted infections (aPR, 0.77; 95% confidence interval [CI], .66–.91) and hygienic reasons (aPR, 0.55; 95% CI, .39–.77) as their motivation to undergo VMMC but were more likely to report being motivated by advice from others (aPR, 1.88; 95% CI, 1.54–2.29). Although most adolescents believed that undergoing VMMC was a normative behavior, younger adolescents were less likely to perceive higher descriptive norms (aPR, 0.79; .71–.89), injunctive norms (aPR, 0.86; 95% CI, .73–1.00), or anticipated stigma for being uncircumcised (aPR, 0.79; 95% CI, .68–.90). Younger adolescents were also less likely than older adolescents to correctly cite that VMMC offers men and boys partial HIV protection (aPR, 0.73; 95% CI, .65–.82). Irrespective of age, adolescents’ main concern about undergoing VMMC was pain (aPR, 0.95; 95% CI, .87–1.04). Among younger adolescents, fear of pain was negatively associated with desire for VMMC (aPR, 0.89; 95% CI, .83–.96).

**Conclusions:**

Age-specific strategies are important to consider to generate sustainable demand for VMMC. Programmatic efforts should consider building on the social norms surrounding VMMC and aim to alleviate fears about pain.

Voluntary medical male circumcision (VMMC) reduces the risk of acquiring human immunodeficiency virus (HIV) by up to 60% [1–5]. The effectiveness of VMMC scale-up, especially in combination with other HIV prevention services, has empirically been shown to be associated with reductions in community-level HIV incidence [6, 7]. Recent mathematical models suggest that increasing and sustaining uptake of VMMC among male adolescents, including 10–14-year-olds, will be critical in maximizing impact of VMMC on the HIV epidemic in sub-Saharan Africa [8]. With a longer sexual history trajectory before them, adolescents stand to gain the most benefit from VMMC uptake—especially if the procedure is conducted before sexual debut. The adolescent VMMC platform also provides an opportunity to expose male adolescents to healthcare settings, engage them in sexual and reproductive healthcare, and ultimately establish positive health promotion practices related to HIV prevention at an early age [9, 10]. Accordingly, the World Health Organization (WHO) and the Joint United Nations Programme on HIV/AIDS (UNAIDS) have set a Fast-Track goal to achieve 90% coverage of VMMC among boys and men aged 10–29 years in high-priority settings in eastern and southern Africa by 2021 [10].

In many regions throughout sub-Saharan Africa, there is already a high demand for VMMC among adolescents [10–12]. Whereas barriers to and facilitators of VMMC uptake have been extensively studied among adult men [13–21], studies among adolescents are limited [22–27]. To the best of our knowledge, no previous study has characterized the decision-making process for VMMC uptake among younger adolescents (aged 10–14 years). Previous studies among adolescents have primarily examined associations with intentions to undergo VMMC, rather than ascertaining the motivations of adolescents presenting for VMMC services—a time when their readiness for the procedure is highest [28]. Thus, it remains unclear what is ultimately driving VMMC demand among male adolescents and what approaches will be necessary to foster and sustain it to maximize HIV epidemic control. In this study, we examine age-specific perceptions of and motivations for VMMC among male adolescents seeking the procedure in three high-priority countries.

## METHODS

### Ethics Statement

This study was approved by the Human Sciences Research Council in South Africa, Tanzania National Institute for Medical Research, Medical Research Council of Zimbabwe, and Johns Hopkins Bloomberg School of Public Health Institutional Review Board. Informed parental consent and adolescent assent was obtained for minors younger than 18 years; adult participants (aged 18 years or older) provided informed consent.

### Study Design

**Figure 1.**
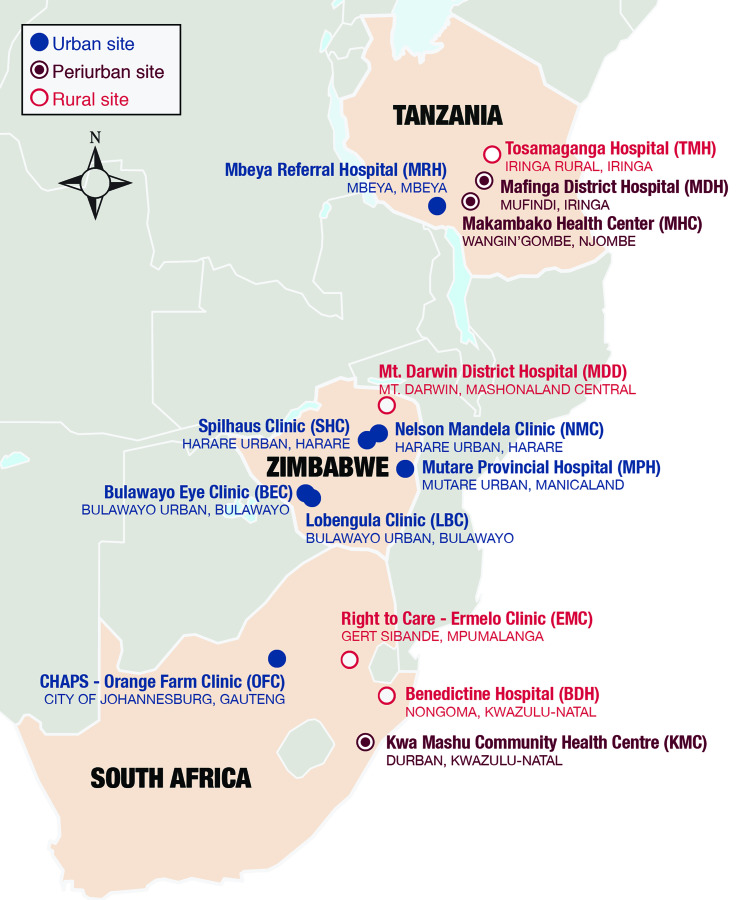
Map of study sites.

From January to September 2016, structured quantitative interviews were conducted with adolescents (aged 10–19 years) seeking VMMC at 14 sites across South Africa, Tanzania, and Zimbabwe (Figure 1). In collaboration with local investigators, national ministries of health, and a technical advisory group, investigators selected study sites using a purposive cluster sampling design. Efforts were made to select health facilities that would be representative of various ethnicities and geographic contexts within each country. Owing to constrained resources, a quota sampling procedure was employed within each country (n = 540), and a convenience sample was interviewed at each site. It should be noted that fewer adolescents were recruited in South Africa (n = 446), partly owing to the holding of a local government election accompanied by community protests and associated restrictions on transportation. Trained research coordinators working with on-site VMMC providers, facility managers, and community mobilizers conducted study recruitment. Lower adolescent client flow was noted at several sites when schools were in session. The study design is further detailed in the Supplementary Materials.

### Data Collection

Before VMMC preprocedure counseling session(s), face-to-face interviews using a structured questionnaire were conducted with adolescents in a private room at the health facility. Interviews were conducted in the adolescents’ local language: Sesotho, isiZulu, or isiSwati (or in English if the participant preferred) in South Africa; KiSwahili in Tanzania; and Shona or Ndebele in Zimbabwe. Research field interviewers were trained on how to conduct interviews, use study instruments, manage survey forms, and enter data. The questionnaire was collaboratively designed with local on-site investigators and the technical advisory group and, when possible, were adapted from instruments previously used in similar contexts [29–32]. The questionnaire was translated from English into the appropriate local language(s).

### Sociodemographic Variables

Age was categorized using standard definitions: 10–14 versus 15–19 years [10]. Hereafter, we refer to the former as “younger adolescents” and the latter as “older adolescents.” The first component of a principal components analysis of self-reported household assets and amenities (eg, tap water, television, motor vehicle) was used to predict a household wealth index (Supplementary Table S1) [33]. An aggregated wealth index was generated and categorized into tertiles to allow comparison of socioeconomic status within the sample. Other sociodemographic information collected included the adolescents’ religion and primary education status. Their sexual history was measured as a dichotomous variable of ever having any type of sexual experience (ie, none versus mutual genital touching, oral, vaginal, or anal sex).

### Motivation to Undergo Voluntary Medical Male Circumcision

#### Level of Desire to Undergo Voluntary Medical Male Circumcision

Adolescents’ level of desire to undergo VMMC [today] was measured on a scale from 0 to 10, with 10 meaning the adolescent definitely wanted to be circumcised. This variable was dichotomized as the highest level of desire (10) versus lower levels (≤9) because the response distribution was skewed. We considered a score ≤9 to be an indication of some level of hesitation about the VMMC procedure.

#### Perceived Motivations to Undergo Voluntary Medical Male Circumcision

To understand adolescents’ perceived motivations to undergo VMMC, participants were asked: “Why are you here to get circumcised today?” Adolescents could provide multiple unprompted responses.

### Voluntary Medical Male Circumcision-Related Perceptions

#### Perceived Descriptive Norms

Perceived descriptive norms refer to perceptions of what people typically do, or the perceived prevalence of a behavior [34, 35]. Perceived descriptive norms to undergo VMMC were measured by the item: “What percentage of your friends do you think are circumcised?” Responses were dichotomized as high (>50%) or low (≤50%).

#### Perceived Injunctive Norms

Perceived injunctive norms involve perceptions of whether a behavior is approved or disapproved [34, 35]. Perceived injunctive norms to undergo VMMC were evaluated by the degree of agreement to 3 items: (1) “Your friends and you encourage each other to get medically circumcised,” (2) “If your friends knew someone was not circumcised, they would encourage him to get circumcised at a medical facility,” and (3) “People in your community are supportive of males your age getting circumcised at a medical facility.” Responses were rated on a Likert scale (from 1 [strongly disagree] to 4 [strongly agree]). Items were summed to form a composite score (α = .67), which was standardized and then dichotomized as high (positive) or low (negative). See Supplementary Table S2 for psychometric details.

#### Anticipated Stigma for Being Uncircumcised

Anticipated stigma (or felt stigma) refers to one’s expectations of negative treatment once a concealed identity is revealed [36]. Anticipated stigma for being uncircumcised was measured using 2 items: (1) “If your friends knew you are not yet circumcised, they would laugh at you” and (2) “If girls knew you are not yet circumcised, they would laugh at you.” Responses were rated on a Likert scale (from 1 [strongly disagree] to 4 [strongly agree]). Items were summed to form a composite score (α = .67), which was standardized and then dichotomized as high (positive) or low (negative). See Supplementary Table S3 for psychometric details.

#### Perceived Level of Human Immunodeficiency Virus Protection From Voluntary Medical Male Circumcision

Adolescents were asked, “Does circumcision protect a male from HIV?” and “Is a circumcised male’s female sex partner protected from HIV?” If the adolescent answered “yes” to either question, he was subsequently prompted to quantify “how much is [a male/his partner] protected?” Responses were coded as 1 (yes, complete protection), 2 (yes, some protection), 3 (no protection), or 4 (don’t know).

#### Concerns About Undergoing Voluntary Medical Male Circumcision

Concerns about undergoing VMMC were examined by the item: “Is there anything that worries you about having the circumcision procedure today?” Multiple unprompted responses were allowed. As a follow-up question, adolescents were asked, “How easy or hard do you expect your recovery to be?” Responses were rated on a Likert scale (from 1 [very easy] to 4 [very hard]).

### Statistical Analysis

The primary independent variable of interest was age group, with older adolescents (aged 15–19 years) as the reference group. Age differences in desire for VMMC, perceived motivations to undergo VMMC, and other VMMC-related perceptions were examined using univariable models and multivariable models that consistently included adjustment for country and setting type. Prevalence ratios were estimated using (population-averaged) modified Poisson regression models with generalized estimating equations and robust standard errors [37, 38]. This approach was selected owing to the high prevalence of each outcome (>10%) and to account for clustering of responses at the health facility level. Because there was a small number of clusters, we also performed sensitivity analyses using a binomial and cluster- specific approach that allowed a random intercept for each site; inferences did not change (data not shown).

As a secondary (exploratory) analysis, we examined characteristics associated with the highest level of desire (10/10) to be circumcised separately among each age group. Age-stratified multivariable models were built by simultaneously adding factors that had an unadjusted association with a high level of desire (10/10) for either age group (*P* < .10) and removing variables that were then insignificant for both age groups (*P* > .05).

Statistical analyses were performed using Stata software, SE version 14.2 (StataCorp, College Station, TX). Statistical significance was examined using Wald tests and 2-sided *P* values. Age-specific data further stratified by country are provided in the Supplementary Materials.

## RESULTS

### Participant Characteristics

The study population included 967 (63.4%) and 559 (36.6%) adolescents who were 10–14 and 15–19 years of age, respectively. Characteristics of the study population are shown stratified by age and country in Table 1 and are further detailed by site in Supplementary Table S4. It was common for adolescents in both age groups to report having a brother they believed was already circumcised (approximately 50%). Comparably, it was less common for adolescents to report having a father they believed was already circumcised, (Table 1). Adolescents reported learning about VMMC for the first time from a variety of sources, which included family members, peers, school officials, health workers, and the media (Table 1).

**Table 1.** Characteristics of the Study Population by Age Group^a^

Characteristic	Adolescents by Country and Age Group, No. (%)							
	South Africa		Tanzania		Zimbabwe		All Countries	
	10–14 y (n = 276)	15–19 y (n = 170)	10–14 y (n = 441)	15–19 y (n = 99)	10–14 y (n = 250)	15–19 y (n = 290)	10–14 y (n = 967)	15–19 y (n = 559)
Setting								
Urban	115 (41.7)	43 (25.3)	189 (42.9)	66 (66.7)	184 (73.6)	209 (72.1)	488 (50.5)	318 (56.9)
Periurban	50 (18.1)	48 (28.2)	128 (29.0)	25 (25.3)	0 (0.0)	0 (0.0)	178 (18.4)	73 (13.1)
Rural	111 (40.2)	79 (46.5)	124 (28.1)	8 (8.1)	66 (26.4)	81 (27.9)	301 (31.1)	168 (30.1)
Primary education								
None	2 (0.7)	7 (4.1)	6 (1.4)	4 (4.0)	1 (0.4)	1 (0.3)	9 (0.9)	12 (2.2)
Incomplete	164 (59.4)	15 (8.8)	414 (93.9)	21 (21.2)	232 (92.8)	182 (62.8)	810 (83.8)	218 (39.0)
Complete	110 (39.9)	146 (85.9)	21 (4.8)	74 (74.8)	17 (6.8)	107 (36.9)	148 (15.3)	327 (58.5)
Household wealth^b^								
Low	7 (2.5)	16 (9.4)	322 (73.0)	70 (70.7)	39 (15.6)	62 (21.4)	368 (38.1)	148 (26.5)
Moderate	112 (40.6)	56 (32.9)	113 (25.6)	24 (24.2)	106 (42.4)	105 (36.2)	331 (34.2)	185 (33.1)
High	157 (56.9)	98 (57.6)	6 (1.4)	5 (5.1)	105 (42.0)	123 (42.4)	268 (27.7)	226 (40.4)
Religion								
Christian	252 (91.3)	155 (91.2)	425 (96.4)	93 (93.9)	239 (95.6)	287 (99.0)	916 (94.7)	535 (95.7)
Muslim	0 (0.0)	2 (1.2)	14 (3.2)	0 (0.0)	5 (2.0)	0 (0.0)	19 (2.0)	2 (0.4)
Traditional	6 (2.2)	9 (5.3)	0 (0.0)	0 (0.0)	0 (0.0)	0 (0.0)	6 (0.6)	9 (1.6)
Agnostic/other	12 (4.3)	4 (2.4)	1 (0.2)	6 (6.1)	6 (2.4)	3 (1.0)	19 (2.0)	13 (2.3)
Ever had sex^c^								
No	258 (93.5)	92 (54.1)	400 (90.7)	44 (44.4)	248 (99.2)	217 (74.8)	906 (93.7)	353 (63.2)
Yes	14 (5.1)	78 (45.9)	41 (9.3)	55 (55.7)	2 (0.8)	73 (25.2)	57 (5.9)	206 (36.9)
Brothers’ MC status								
Circumcised^d^	159 (57.6)	96 (56.5)	252 (57.1)	65 (65.7)	75 (30.0)	77 (26.6)	486 (50.3)	238 (42.6)
Uncircumcised	71 (25.7)	38 (22.4)	38 (8.6)	9 (9.1)	99 (39.6)	120 (41.4)	208 (21.5)	167 (29.9)
Don’t know	7 (2.5)	1 (0.6)	13 (3.0)	3 (3.0)	2 (0.8)	11 (3.8)	22 (2.3)	15 (2.7)
No brother	39 (14.1)	35 (20.6)	138 (31.3)	22 (22.2)	74 (29.6)	82 (28.3)	251 (26.0)	139 (24.9)
Father’s MC status								
Circumcised	111 (40.2)	34 (20.0)	197 (44.7)	33 (33.3)	59 (23.6)	39 (13.5)	367 (38.0)	106 (19.0)
Uncircumcised	43 (15.6)	41 (24.1)	27 (6.1)	13 (13.1)	94 (37.6)	107 (36.9)	164 (17.0)	161 (28.8)
Don’t know	95 (34.4)	63 (37.1)	199 (45.1)	44 (44.4)	68 (27.2)	67 (23.1)	362 (37.4)	174 (31.1)
Deceased father	26 (9.4)	30 (17.7)	18 (4.1)	9 (9.1)	29 (11.6)	77 (26.6)	73 (7.6)	116 (20.8)
VMMC information source^e^								
Parent	19 (6.9)	10 (5.9)	52 (11.8)	2 (2.0)	8 (3.2)	6 (2.1)	79 (8.2)	18 (3.2)
Other family	18 (6.5)	12 (7.1)	47 (10.7)	7 (7.1)	12 (4.8)	4 (1.4)	77 (8.0)	23 (4.1)
Peers	38 (13.8)	21 (12.4)	96 (21.8)	22 (22.2)	16 (6.4)	28 (9.7)	150 (15.1)	71 (12.7)
School	99 (35.9)	62 (36.5)	99 (22.5)	6 (6.1)	139 (55.6)	135 (46.6)	337 (34.9)	203 (36.3)
Health worker^f^	89 (32.3)	51 (30.0)	128 (29.0)	45 (45.5)	61 (24.4)	94 (32.4)	278 (28.8)	190 (34.0)
Media	10 (3.6)	10 (5.9)	8 (1.8)	14 (14.1)	7 (2.8)	15 (5.2)	25 (2.6)	39 (7.0)
Other	3 (1.1)	4 (2.4)	5 (1.1)	0 (0.0)	5 (2.0)	3 (1.0)	13 (1.3)	7 (1.3)

Abbreviations: MC, male circumcision; VMMC, voluntary medical male circumcision.

^a^Proportions may not add up to 100% owing to missing data.

^b^Distribution of wealth score based on household assets and amenities.

^c^Any kind of sexual experience (mutual genital touching or oral, vaginal, or anal sex).

^d^The adolescent is aware that at least 1 brother has been circumcised.

^e^The initial source from which participants remember first learning about VMMC.

^f^Including health facility workers, community health workers, and VMMC mobilizers.

### Age Differences in the Desire and Perceived Motivations to Undergo Voluntary Medical Male Circumcision

A high proportion of younger (72.6%) and older (72.1%) adolescents reported a 10/10 desire for VMMC, and these proportions did not differ significantly by age group (adjusted prevalence ratio [aPR], 1.01; 95% confidence interval [CI], .97–1.06; *P* = .50). Younger and older adolescents reported many reasons for wanting to undergo VMMC (Table 2). The most common reason among each group was protection from HIV or other sexually transmitted infections (STIs), but this was cited less frequently among younger adolescents than older adolescents (58.8% vs 83.9%; aPR, 0.77; 95% CI, .66–.91; *P* = .002; Table 2). Younger adolescents were less motivated than older adolescents to undergo VMMC for hygienic reasons (15.7% vs 28.3%; aPR, 0.55; 95% CI, .39–.77; *P* < .001; Table 2). On the other hand, younger adolescents were more likely to report external cues as reasons for seeking VMMC, such as suggestions they received from school officials (6.1% vs 1.6%; aPR, 1.98; 95% CI, .98–3.99; *P* = .056) and advice from others (28.9% vs 9.8%; aPR, 1.88; 95% CI, 1.54–2.29; *P* < .001; Table 2). A small proportion of both age groups (<10%) acknowledged they were seeking VMMC because their “friends were doing it” or “to avoid stigma, shame, or ridicule” (Table 2). Variations in perceived motivations by age and country are shown in Supplementary Table S5.

**Table 2.** Age Differences in Perceived Motivations to Undergo Voluntary Medical Male Circumcision^a^

Reason for Undergoing VMMC	Adolescents, No. (%)		PR (95% CI)	aPR (95% CI)
	Aged 10–14 y (n = 967)	Aged 15–19 y (n = 559)		
Someone advised it (eg, parent)	279 (28.9)	55 (9.8)	1.86 (1.49–2.33)^b^	1.88 (1.54–2.29)^b^
Suggested by school	59 (6.1)	9 (1.6)	1.81 (1.23–2.65)^b^	1.98 (0.98–3.99)
Want to be healthy	82 (8.5)	69 (12.3)	0.60 (.45–.81)^b^	0.59 (.43–.80)^b^
To protect myself from HIV/STIs	569 (58.8)	469 (83.9)	0.77 (.67–.90)^b^	0.77 (.66–.91)^b^
To protect myself/partner from cancer	12 (1.2)	30 (5.4)	0.46 (.27–.79)^b^	0.38 (.20–.73)^b^
To improve hygiene/easier to clean	152 (15.7)	158 (28.3)	0.56 (.41–.77)^b^	0.55 (.39–.77)^b^
Make my penis more attractive	25 (2.6)	17 (3.0)	0.77 (.37–1.57)	0.66 (.28–1.59)
Heard sex will be better	4 (0.4)	20 (3.6)	0.12 (.05–.25)^b^	0.10 (.05–.22)^b^
Friends were doing/did it	95 (9.8)	43 (7.7)	0.94 (.73–1.23)	0.87 (.68–1.11)
To become a man/adult	2 (0.2)	7 (1.3)	0.19 (.06–.54)^b^	0.17 (.03–.90)^b^
To avoid stigma, shame, or ridicule	18 (1.9)	10 (1.8)	0.65 (.26–1.62)	0.52 (.17–1.63)

Abbreviations: aPR, adjusted prevalence ratio; CI, confidence interval; HIV, human immunodeficiency virus; PR, prevalence ratio; STIs, sexually transmitted infections; VMMC, voluntary medical male circumcision.

^a^Participants could provide multiple (unprompted) responses. PRs were estimated by modified Poisson regression with generalized estimating equations and robust standard errors to account for clustering of responses at the facility level; aPRs were estimated from multivariable models including adjustment for country and facility setting. The reference group was older adolescents (aged 15–19 years).

^b^ *P* < .05.

### Age Differences in Voluntary Medical Male Circumcision-Related Perceptions

#### Perceived Norms and Anticipated Stigma

**Figure 2.**
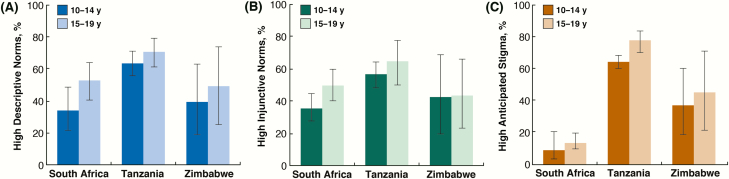
Age and country differences in perceived norms of voluntary medical male circumcision (VMMC) and anticipated stigma for being uncircumcised. High descriptive norms refers to the perception that >50% of the adolescents’ friends were circumcised. High injunctive norms refers to the perception that VMMC is an approved behavior. High anticipated stigma refers to expectations of negative treatment if peers found out that the adolescent was not yet circumcised. Error bars represent design-based 95% confidence intervals as estimated by Taylor series linearization to account for clustering at the health facility level.

More than one-half of the study population (median, 60%; interquartile range, 20%–90%) believed that the majority (>50%) of their friends were circumcised. Among all study participants, age, country, and setting type were independently associated with high descriptive VMMC norms, injunctive VMMC norms, and anticipated stigma for being uncircumcised (Supplementary Table S6). In particular, younger adolescents were less likely than older ones to report high descriptive VMMC norms (49.0% vs 53.9%; aPR, 0.79; 95% CI, .71–.89; *P* < .001), high injunctive VMMC norms (46.7% vs 49.1%; aPR, 0.86; 95% CI, .73–1.00; *P* = .052), and anticipated stigma for being uncircumcised (41.6% vs 41.3%; aPR, 0.79; 95% CI, .68–.90; *P* = .001). Although high descriptive norms and anticipated stigma for being uncircumcised were associated with age within all 3 countries (Figure 2A and 2C), high perceived injunctive norms seemed to be associated with age in only South Africa and Tanzania (Figure 2B). Of note, anticipated stigma for being uncircumcised was lowest in South Africa among both age groups (Figure 2C).

#### Perceived Level of Human Immunodeficiency Virus Protection From Voluntary Medical Male Circumcision

**Figure 3.**
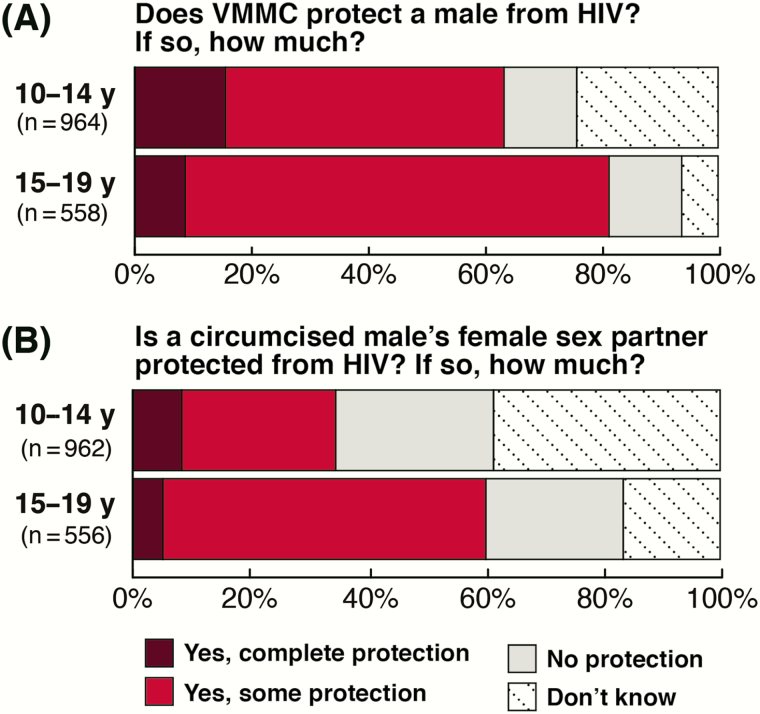
Age differences in the perceived level of human immunodeficiency virus protection provided by voluntary medical male circumcision.

Age was associated with perceived level of HIV protection from VMMC (Figure 3). Younger adolescents were less likely than older ones to correctly cite that VMMC offers boys and men only “some” protection from HIV (47.8% vs 72.6%; aPR, 0.73; 95% CI, .65–.82; *P* < .001). Younger adolescents (15.3%) were almost twice as likely than older adolescents (8.8%) to misperceive VMMC as offering them complete protection from HIV. All adolescents had poor knowledge regarding the level of HIV protection VMMC offers to female partners (Figure 3B). Only 27.0% of younger and 23.4% of older adolescents correctly cited that VMMC does not directly provide female partners HIV protection (aPR, 1.04; 95% CI, .86–1.26; *P* = .71). Notably, 34.1% of younger adolescents and 59.1% of older adolescents misperceived that female partners had some or complete HIV protection after VMMC (Figure 3B). These data are shown by age and country in Supplementary Table S7.

#### Perceived Barriers to Undergoing Voluntary Medical Male Circumcision

The most common concern about undergoing VMMC was pain from the procedure or injection site among younger (44.5%) and older (66.4%) adolescents. Concerns about pain, however, were not independently associated with age (aPR, 0.95; 95% CI, .87–1.04; *P* = .27) (Table 3). Other less frequent concerns were related to the duration of postprocedure wound healing time, sexual abstinence during wound healing, and potential damage to the penis (Table 3). In addition, 13.1% of younger and 8.4% of older adolescents expected that their recovery would be “hard” or “very hard” (aPR, 1.44; 95% CI, 1.12–1.86; *P* = .004). Perceived barriers to undergoing VMMC are shown by age and country in Supplementary Table S8.

**Table 3.** Age Differences in Concerns About Undergoing Voluntary Medical Male Circumcision^a^

Perceived Concern	Adolescents, No. (%)		PR (95% CI)	aPR (95% CI)
	Aged 10–14 y (n = 967)	Aged 15–19 y (n = 559)		
Pain from procedure/injection	430 (44.5)	371 (66.4)	0.95 (.87–1.03)	0.95 (.87–1.04)
Duration of healing time	22 (2.3)	25 (4.5)	0.64 (.27–1.50)	0.71 (.28–1.78)
Sexual abstinence during wound healing^b^	5 (0.5)	13 (2.3)	0.22 (.09–.62)^c^	…
Potential damage to penis	11 (1.1)	13 (2.3)	0.49 (.22–1.08)	0.67 (.30–1.51)

Abbreviations: aPR, adjusted prevalence ratio; CI, confidence interval; PR, prevalence ratio.

^a^Participants could provide multiple responses (unprompted). PRs were estimated by modified Poisson regression with generalized estimating equations and robust standard errors to account for clustering of responses at the facility level; aPRs were estimated from multivariable models that included adjustment for country and facility setting. The reference group was older adolescents (aged 15–19 years).

^b^Response was observed only in South Africa. The multivariable model failed to converge, so there is no corresponding adjusted estimate.

^c^ *P* < .05.

### Factors Associated With Desire to Undergo Voluntary Medical Male Circumcision by Age Group

Age-specific correlates of the highest level of desire (10/10) to be circumcised are shown in Table 4. Among younger adolescents, high perceived injunctive norms (aPR, 1.07; 95% CI, 1.03–1.12; *P* < .001) and high anticipated stigma (aPR, 1.04; 95% CI, 1.02–1.07; *P* < .001) were positively associated with high levels of desire to be circumcised, whereas concern about pain was negatively associated with a high level of desire to be circumcised (aPR, 0.89; 95% CI, .83–.96; *P* = .002; Table 4). Interestingly, these associations were not detected among older adolescents (Table 4). Although a higher perceived level of HIV protection for boys and men undergoing VMMC was not independently associated with a high level of desire to be circumcised among either age group (Table 4), both younger (aPR, 1.08; 95% CI, 1.01–1.16; *P* = .03) and older (aPR, 1.05; 95% CI, .998–1.10; *P* = .06) adolescents who misperceived that VMMC offered female partners complete HIV protection were more likely to have a high level of desire to be circumcised (Table 4).

**Table 4.** Factors Associated With the Highest Level of Desire to Undergo Voluntary Medical Male Circumcision^a^

Factor	Adolescents Aged 10–14 y			Adolescents Aged 15–19 y		
	% (No./Total No.)	PR (95% CI)	aPR (95% CI)	% (No./Total No.)	PR (95% CI)	aPR (95% CI)
Country						
South Africa	53.3 (147/276)	Reference	Reference	60.6 (103/170)	Reference	Reference
Tanzania	83.0 (366/441)	1.22 (1.12–1.33)^b^	1.15 (1.11–1.20)^b^	97.0 (96/99)	1.25 (1.13–1.38)^b^	1.22 (1.12–1.32)^b^
Zimbabwe	75.6 (189/250)	1.15 (.98–1.36)^c^	1.20 (1.04–1.38)^b^	70.3 (204/290)	1.04 (.87–1.24)	1.09 (.95–1.24)
Setting						
Urban	75.2 (367/488)	Reference	Reference	71.7 (228/318)	Reference	Reference
Periurban	80.3 (143/178)	1.05 (.90–1.22)	1.01 (.96–1.07)	86.3 (63/73)	1.17 (1.00–1.35)^b^	1.12 (1.01–1.23)^b^
Rural	63.8 (192/301)	0.94 (.78–1.13)	0.96 (.88–1.05)	66.7 (112/168)	0.99 (.83–1.19)	1.01 (.90–1.14)
Primary education						
None or incomplete	73.9 (605/819)	Reference	…	76.1 (175/230)	Reference	…
Completed	65.5 (97/148)	1.02 (.97–1.07)	…	69.4 (227/327)	0.98 (.92–1.04)	…
Household wealth						
Low	71.0 (233/328)	Reference	…	71.3 (134/188)	Reference	…
Moderate	73.2 (259/354)	1.00 (.98–1.02)	…	71.3 (159/223)	1.02 (.98-1.07)	…
High	73.7 (210/285)	0.98 (.94–1.01)	…	74.3 (110/148)	1.00 (.96–1.05)	…
Religion						
Christian	72.8 (667/916)	Reference	…	72.2 (386/535)	Reference	…
Muslim	79.0 (15/19)	1.02 (.91–1.13)	…	100.0 (2/2)	1.34 (1.23–1.46)^b^	…
Traditional	66.7 (4/6)	1.05 (.76–1.46)	…	55.6 (5/9)	1.02 (.90–1.15)	…
Agnostic/other	52.6 (10/19)	0.98 (.84–1.15)	…	76.9 (10/13)	0.99 (.88–1.12)	…
Ever had sex (any)						
No	72.3 (655/906)	Reference	…	72.0 (254/353)	Reference	…
Yes	80.7 (46/57)	1.02 (.95–1.09)	…	72.3 (149/206)	1.00 (.96–1.05)	…
Circumcised brother						
No	71.5 (344/481)	Reference	…	72.0 (231/321)	Reference	…
Yes	73.7 (358/486)	1.01 (.97–1.06)	…	72.3 (172/238)	0.99 (.96–1.03)	…
Circumcised father						
No	70.8 (424/599)	Reference	…	72.5 (327/451)	Reference	…
Yes	75.5 (277/367)	1.04 (1.00–1.07)^b^	…	70.8 (75/105)	0.99 (.94–1.03)	…
Descriptive norms						
Low	65.6 (318/485)	Reference	…	71.1 (182/256)	Reference	…
High	80.5 (375/466)	1.06 (1.00–1.12)^c^	…	73.6 (220/299)	0.99 (.93–1.06)	…
Injunctive norms						
Low	64.4 (322/500)	Reference	Reference	69.3 (196/283)	Reference	Reference
High	83.1 (364/438)	1.08 (1.03–1.13)^b^	1.07 (1.03–1.12)^b^	75.1 (205/273)	1.01 (.97–1.05)	1.01 (.96–1.05)
Anticipated stigma						
Low	63.7 (353/554)	Reference	Reference	63.2 (206/326)	Reference	Reference
High	86.6 (341/394)	1.06 (1.03–1.10)^b^	1.04 (1.02–1.07)^b^	84.7 (194/229)	1.02 (.997–1.04)^c^	1.01 (.98–1.03)^c^
HIV protection among males						
None	64.1 (75/117)	Reference	…	73.5 (50/68)	Reference	…
Yes, some	74.6 (344/461)	1.02 (.93–1.11)	…	72.8 (295/405)	0.97 (.90–1.05)	…
Yes, completely	76.9 (113/147)	1.08 (1.01–1.15)^b^	…	65.3 (32/49)	0.97 (.90–1.05)	…
Don’t know	71.1 (170/239)	1.02 (.95–1.10)	…	72.2 (26/36)	0.98 (.87–1.10)	…
HIV protection among females						
None	77.2 (61/79)	Reference	Reference	70.8 (92/130)	Reference	Reference
Yes, some	74.3 (185/249)	1.03 (.99–1.06)	1.04 (1.01–1.08)^b^	70.6 (216/306)	1.00 (.93–1.07)	1.00 (.92–1.07)
Yes, completely	69.6 (181/260)	1.08 (1.01–1.16)^b^	1.08 (1.01–1.16)^b^	88.5 (23/26)	1.06 (1.01–1.11)^b^	1.05 (.998–1.10)^c^
Don’t know	72.7 (272/374)	1.03 (.99–1.07)	1.05 (.996–1.11)^c^	73.4 (69/94)	1.01 (.95–1.07)	1.00 (.94–1.07)
Concerned about pain						
No	80.6 (433/537)	Reference	Reference	79.8 (150/188)	Reference	Reference
Yes	62.6 (269/430)	0.88 (.83–.95)^b^	0.89 (.83–.96)^b^	68.2 (253/371)	0.99 (.94–1.04)	1.00 (.95–1.05)
Expected recovery						
Easy/very easy	74.6 (620/831)	Reference	…	73.6 (376/511)	Reference	…
Hard/very hard	60.0 (75/125)	0.94 (.88–.99)^b^	…	57.5 (27/47)	0.99 (.88–1.11)	…

Abbreviations: aPR, adjusted prevalence ratio; CI, confidence interval; HIV, human immunodeficiency virus; PR, prevalence ratio.

^a^The outcome was the highest level of desire (10/10) to be circumcised the same day. PRs were estimated by modified Poisson regression with generalized estimating equations and robust standard errors to account for clustering of responses at the facility level. aPRs were estimated from age-stratified multivariable models that included covariates that significantly contributed to either age-stratified model, as indicated by global Wald tests and a significance threshold of .05. The reference group was older adolescents (aged 15–19 years). There was no significant multicollinearity observed in the models presented. Results from this exploratory analysis, particularly the effect sizes, should be interpreted with caution as there was limited variability in the outcome.

^b^ *P* < .05.

^c^ *P* < .10.

## DISCUSSION

VMMC is a key biomedical intervention that can improve the overall health of male adolescents in sub-Saharan Africa, while contributing to HIV epidemic control. This study provides quantitative evidence that VMMC is often perceived to be a normative behavior among adolescents seeking the procedure in 3 countries. Many adolescents feel they are expected to be circumcised, as indicated by high perceived injunctive norms and anticipated stigma from peers and girls. This may partly explain the high natural demand for VMMC among adolescents [10]. Compared with older adolescents, younger adolescents were less concerned about the stigma of being uncircumcised and less likely to seek VMMC for HIV/STI protection or hygiene reasons. Instead, younger adolescents were more likely to report seeking VMMC because of external cues from others. Understanding these age-specific factors is important for VMMC programs that continue to scale up in some settings while transitioning to a phase of sustainability in others.

Several findings of this study are consistent with those of previous studies. Similar to other studies of older adolescents and adults [10, 13, 14, 17–22], pain was the most common concern among adolescents seeking VMMC in our study—regardless of age. For purposes of demand generation and as an essential component of preprocedure counseling, it needs to be clearly communicated to adolescents that, in comparison with traditional circumcision, VMMC is a minor surgical operation performed by medical staff with the use of anesthesia. Concerns over possible infertility, penile injury, and delay in sexual intercourse during wound healing highlighted in studies of older adolescents and adults [10, 13, 14, 17, 18], were less commonly cited by younger adolescents in this study.

The high proportion of adolescents motivated to seek VMMC, coupled with supportive adjoining social norms, is encouraging and to be expected given the maturity of the VMMC program across the focus countries studied. Although the most common reason for seeking VMMC among all adolescents regardless of age was protection from HIV/STIs, younger adolescents were far more likely to believe that VMMC offers complete protection, rather than knowing the accurate figure of approximately 60% protection [1–3]. Furthermore, three-quarters of all adolescents incorrectly believed that VMMC directly offers HIV protection to female partners. These misperceptions, particularly among younger adolescents aged 10–14 years, are concerning and will require age-appropriate counseling to ensure a clear understanding of the benefits of VMMC and to prepare adolescents for a lifetime of HIV-preventive behaviors once they become sexually active.

This study has several limitations. First, as a cross-sectional study, all reported associations are descriptive and cannot be interpreted in a causal manner. Second, there is potential for selection bias. All adolescents in this study were in the “action” stage of readiness for VMMC, according to the transtheoretical model [39], and their beliefs or perceptions of VMMC may or may not differ from those held by adolescents in earlier stages of readiness. Although we were limited by the number of sites per country and the use of convenience sampling at each site, the consistency in age differences observed across multiple sites supports the generalizability of our findings. Finally, it should be reiterated that our age-stratified analysis of factors associated with desire to undergo VMMC was meant to be exploratory, given that desire was measured by a single item, which also had a limited range and skewed distribution.

Successfully generating demand for VMMC among adolescents has required substantial effort, but sustaining this demand will probably require continued roll-out of strategic public health programs [10]. Building on social norms surrounding VMMC while alleviating concerns about pain may be useful strategies to increase or sustain demand for VMMC in this key population. The data in this study indicate an urgent need to implement educational initiatives to dispel misperceptions of VMMC in the community and appropriately counsel adolescents and their parents/guardians before the procedure [40]. Finally, additional work is needed to clarify how adolescents make decisions concerning VMMC in order to implement effective, youth-centered strategies to generate demand for the procedure.

## Supplementary Data

Supplementary materials are available at *Clinical Infectious Diseases* online. Consisting of data provided by the authors to benefit the reader, the posted materials are not copyedited and are the sole responsibility of the authors, so questions or comments should be addressed to the corresponding author.

Supplementary_Tables

## Notes

***Acknowledgments.*** The authors are sincerely grateful to the adolescent participants in this study for sharing their perspectives and experiences. We would also like to thank the facility managers, community mobilizers, and providers for their support. Furthermore, we would like to acknowledge the contributions of the Technical Advisory Group for the adolescent VMMC assessment and their guidance throughout the study. We are privileged to work with this group of dedicated professionals from the US President’s Emergency Plan for AIDS Relief (PEPFAR), the US Centers for Disease Control and Prevention, the US Department of Defense, the World Health Organization, United Nations Children’s Fund, and the Bill & Melinda Gates Foundation. We are thankful to the Human Sciences Research Council in South Africa, CSK Research Solutions in Tanzania, PSI/Zimbabwe, and the Centre for Sexual Health & HIV/AIDS Research in Zimbabwe for assistance with data collection. We also appreciate the assistance of Meaghen Murphy and Zoe R. Packman with copyediting and Maria Elena Figueroa with project start-up.

***Financial support.*** This work was supported by the United States Agency for International Development with PEPFAR funding (cooperative agreement AID-OAA-A-12-00058) to the Johns Hopkins Center for Communication Programs and cofunded by the UK Department of International Development through the Integrated Support Program in Zimbabwe.

***Supplement sponsorship.*** This article appears as part of the supplement “Adolescent Voluntary Medical Male Circumcision: Vital Intervention Yet Improvements Needed,” sponsored by Johns Hopkins University.

***Potential conflicts of interest.*** All authors: No reported conflicts of interest. All authors have submitted the ICMJE Form for Disclosure of Potential Conflicts of Interest. Conflicts that the editors consider relevant to the content of the manuscript have been disclosed.
