# Supplementary material for: Age Differences in Perceptions of and Motivations for Voluntary Medical Male Circumcision Among Adolescents in South Africa, Tanzania, and Zimbabwe
Source: Clin Infect Dis. 2018 Apr 3;66(Suppl 3):S173–82. doi: 10.1093/cid/cix951 (PMC5888947; doi:10.1093/cid/cix951)
Supplement: Supplementary_Tables [file cix951_suppl_patel_motivation_supplementalmaterial_final.pdf]

SUPPLEMENTARY MATERIAL

Addendum to methods: study design.
Supplementary Table 1. Principal components analysis of household-level socioeconomic status indicators.
Supplementary Table 2. Principal components analysis of items measuring injunctive perceived VMMC norms.
Supplementary Table 3. Principal components analysis of items measuring anticipated stigma for being uncircumcised.
Supplementary Table 4. Sociodemographic characteristics of the study population stratified by health facility.
Supplementary Table 5. Perceived motivations to undergo VMMC stratified by age group and country.
Supplementary Table 6. Association of age group and perceived VMMC norms and anticipated stigma.
Supplementary Table 7. Perceived level of HIV protection from VMMC stratified by age group and country.
Supplementary Table 8. Perceived concerns about undergoing VMMC stratified by age group and country.

Study Design Addendum:

Selection of VMMC Study Sites

Study sites in each country were selected to represent a wide range of voluntary medical male circumcision (VMMC) facilities based on the following criteria: area (urban, periurban, and rural), ethnic groups, type of clinic (fixed, mobile, or both), governance and volume of clients (low ≤ 30 clients or high > 30 clients). In Tanzania, ethnic diversity was considered in site selection. In South Africa, study site selection also considered knowledge, culture, and practices regarding VMMC, thereby selecting provinces that represent this diversity in regards to VMMC knowledge and practice. Geographical and ethnic contexts were also considered in Zimbabwe, in addition to clinics that provided both VMMC and linkages to adolescent, sexual and reproductive health (ASRH) services.

Characteristics of VMMC Study Sites

Site	Setting *District/Region*	Area	Type *Fixed/mobile*	Governance	Population *Language(s)**	Volume *Clients*
**Tanzania**						
Mafinga District Hospital	Mufindi/Iringa	Periurban	Fixed	Public	Hehe (Swahili), serves timber plantations	Low
Makambako Health Center	Wanging'ombe/ Njombe	Periurban	Fixed	Public	Wabena (Swahili)	High
Mbeya Referral Hospital	Mbeya/Mbeya	Urban	Both	Public	Shona (Swahili)	High
Tosamaganga Hospital	Iringa Rural/Iringa	Rural	Fixed	Public	Hehe (Swahili), serves mostly adolescents	Low
**South Africa**						
Benedictine Hospital (MaTCH)	Nongoma/ Kwazulu-Natal	Rural	Mobile	NGO	isiSwati, isiZulu	Low
Ermelo Hospital (Right to Care)	Gert Sibande/ Mpumalanga	Rural	Fixed	NGO	isiZulu	High
KwaMashu Community Health Centre (MaTCH)	Durban/ Kwazulu-Natal	Periurban	Fixed	Public	isiZulu	Low
Orange Farm Clinic (CHAPS)	Johannesburg/ Guateng	Urban	Fixed	NGO	Sesotho, isiZulu; serves mostly adolescents and clients from other areas	High
**Zimbabwe**						
Bulawayo Eye Clinic	Bulawayo Urban/Bulawayo	Urban	Fixed	NGO	Ndebele	High
Lobengula Clinic	Bulawayo Urban/Bulawayo	Urban	Fixed	Mixed	Ndebele	High
Mt. Darwin District Hospital	Mt. Darwin/ Mashonaland Central	Rural	Both	Public	Shona	Low
Mutare Provincial Hospital	Mutare Urban, Manicaland	Urban	Fixed	Public	Manica, Shona	Low
138 Nelson Mandela Clinic	Harare Urban, Harare	Urban	Fixed	NGO	Shona	High
Spillhaus Clinic	Harare Urban, Harare	Urban	Fixed	Public	Shona	High

Notes:
a.) Non-governmental organization (NGO)
b.) Mixed refers to both public and private governance
c.) *South Africa lists languages only

Dates of Data Collection

Country	Quantitative Surveys
**South Africa**	Feb. 2016 – Sept. 2016
**Tanzania**	Jan. 2016 – Mar. 2016
**Zimbabwe**	Feb. 2016 – Sept. 2016

Notes:
a.) Quantitative includes pre- and post-procedure surveys with male adolescents (10-19 years) who had agreed to receive VMMC.

Recruitment and Consent of Study Participants

In all three countries, recruitment for the study was conducted in collaboration with selected VMMC facilities, NGOs, and governmental public health and educational institutions (schools). The study utilized VMMC mobilizers and research staff to recruit participants. VMMC mobilizers play a central role in recruiting male adolescents and older males to undergo VMMC in the three countries. They are generally associated with NGOs and work closely with health facilities and schools. In the case of in-school mobilization, VMMC mobilizers discuss VMMC with male adolescents and accompany those interested to their homes and discuss the minor's VMMC decision with parents to obtain consent for the procedure. VMMC providers also recruited adolescents who agreed to be circumcised. All recruiters used recruitment scripts to inform participants about the study and invite them to participate.

Recruitment and consenting process with male adults. In all countries, males were approached with information regarding the study by recruiters. If they were interested in participating, adult consent was obtained. VMMC mobilizers informed male adolescents ages 18-19 who had agreed to be circumcised about the study and invited them for the interview on the day of their VMMC.

Recruitment and consenting process with male minors (under age 18). In all countries, for minors (10-17 years), mobilizers first informed parents/guardians about the study and then the children of those parents who agreed to have their son be part of the study. Some minors were recruited at clinics at the time of the VMMC where VMMC providers and the study team approached them on the day of the VMMC. Accompanying parents/guardians provided consent. In Tanzania, parents who did not accompany their children were contacted by phone to inform them about the study and obtain oral consent by phone.

Data Storage, Management, and Quality Assurance

In each country, a local senior co-investigator, monitored daily activities and monitored data security. Data collection forms were coded with study participants' study ID numbers. Codes and links between study IDs and identifiers were stored securely in a separate place (locked storage cabinet or secure electronic database). The data were stored on a secure server protected by limited access and strong password systems.

Quantitative data collection was conducted using electronic handheld tablets in Zimbabwe and laptops in Tanzania. Paper questionnaires were used in South Africa and subsequently processed electronically. All electronic files for the quantitative survey database were de-identified (personal identifiers were removed) and password protected in the local country before delivering it to the study team in Baltimore.

Quality assurance measures were implemented throughout data collection. Quality checks on data collected or entered using electronic tablets/laptops or data entry software were conducted, including but not limited to validation of skip patterns, coding of response categories, and checking of inconsistent, refusal, or blank responses. A master codebook of all variables and response categories was created and validated against the incoming data in all countries. In each country, the data were checked and monitored by local data managers and/or coordinators In addition to receiving weekly data collection reports in all countries during data collection, the study team in Baltimore, MD held weekly meetings with local study teams to discuss ongoing activities and coordination during data collection.

Supplementary Table 1. Principal components analysis of household-level socioeconomic status indicators (N=1526).

Household-Level SES Indicators	South Africa (n=446)		Tanzania (n=540)		Zimbabwe (n=540)		All Countries (n=1526)	
	Mean (SD)	Factor Score	Mean (SD)	Factor Score	Mean (SD)	Factor Score	Mean (SD)	Factor Score
**Water source**								
Piped into dwelling	0.26 (0.44)	0.230	0.43 (0.49)	0.292	0.41 (0.49)	0.203	0.52 (0.50)	0.116
Piped to yard/plot	0.56 (0.50)	0.099	0.11 (0.32)	0.014	0.22 (0.41)	-0.007	0.04 (0.20)	0.132
Public tap/neighbor's tap/standpipe	0.09 (0.28)	-0.170	0.17 (0.38)	-0.150	0.14 (0.34)	0.051	0.14 (0.35)	-0.066
Tube well or borehole	0.01 (0.09)	-0.086	0.10 (0.31)	-0.135	0.06 (0.24)	-0.140	0.06 (0.24)	-0.153
Dug well	0.09 (0.29)	-0.324	0.12 (0.32)	-0.086	0.15 (0.36)	-0.199	0.23 (0.42)	-0.094
River/stream/natural spring	0.00 (0.00)	-	0.07 (0.25)	-0.093	0.02 (0.15)	-	0.00 (0.00)	-0.126
**Sanitation facility**								
Flushed or pour flush toilet	0.63 (0.48)	0.488	0.44 (0.50)	0.383	0.63 (0.48)	0.427	0.81 (0.39)	0.361
Covered pit latrine	0.29 (0.45)	-0.381	0.26 (0.44)	-0.131	0.24 (0.43)	-0.412	0.18 (0.38)	-0.204
Open pit latrine /no facility	0.08 (0.27)	-0.234	0.30 (0.46)	-0.290	0.13 (0.34)	-0.091	0.02 (0.13)	-0.258
**Durable assets**								
Electricity	0.93 (0.25)	0.366	0.28 (0.45)	0.430	0.64 (0.48)	0.415	0.76 (0.42)	0.430
Radio	0.84 (0.37)	0.093	0.75 (0.43)	0.057	0.80 (0.40)	0.162	0.81 (0.39)	0.146
Television	0.94 (0.23)	0.264	0.22 (0.41)	0.437	0.65 (0.48)	0.371	0.85 (0.36)	0.429
Home telephone	0.15 (0.35)	-0.001	0.01 (0.10)	0.024	0.08 (0.27)	0.128	0.10 (0.30)	0.146
Mobile phone	0.91 (0.29)	0.126	0.89 (0.31)	0.197	0.92 (0.28)	0.137	0.95 (0.22)	0.108
Refrigerator	0.90 (0.30)	0.351	0.03 (0.17)	0.286	0.51 (0.50)	0.363	0.66 (0.47)	0.415
**Transportation**								
Bicycle	0.30 (0.46)	0.040	0.52 (0.50)	0.037	0.39 (0.49)	-0.011	0.33 (0.47)	-0.083
Motorcycle	0.07 (0.26)	0.048	0.16 (0.37)	0.116	0.10 (0.30)	0.046	0.06 (0.24)	-0.023
Car/truck/mini-van	0.64 (0.48)	0.072	0.05 (0.22)	0.308	0.34 (0.47)	0.179	0.37 (0.48)	0.295
**Eigenvalue**		2.75		2.92		4.07		3.77
**Percent of Variance Explained**		16.2%		16.2%		24.0%		21.0%

The unstandardized mean response for possession of each household level indicator and its standard deviation (SD) are shown. Each response option for nominal categorical variables (i.e., water source and sanitation facility) was dichotomized. Principal components analysis was conducted among the overall sample. The overall unrotated factor scores of the first component were used to predict the participants' household wealth score for "all countries". The "all countries" wealth score was categorized into tertiles for the primary analysis to allow estimated comparisons of household wealth within the sample population. We also performed the principal components analysis separately for each individual country to confirm factor scores were similar. The data shown above also includes 15 participants who were initially missing data for ≥1 item. For missing items among these 15 participants, we imputed the most common response provided at the facility the participant attended. Imputation did not appreciably change the factor scores.

<u>Supplementary Table 2.</u> Principal components analysis of items measuring injunctive perceived VMMC norms (N=1494).

Items	Mean (SD)	Factor Loading	Communality Estimate
Your friends and you encourage each other to get medically circumcised.	3.20 (0.72)	0.8667	0.7569
If your friends knew someone was not circumcised, they would encourage him to get circumcised at a medical facility	3.15 (0.73)	0.8572	0.7348
People in your community are supportive of males your age getting circumcised at a medical facility.	3.32 (0.61)	0.7146	0.5107

Responses to each item included 1-strongly disagree, 2-disagree, 3-agree, 4-strongly agree. The unstandardized mean response to each item and its standard deviation (SD) are presented—data are only shown among complete cases. Following principal component analysis (PCA) using a polychoric correlation structure, several criteria were used to confirm that the items were unidimensional: (1) eigenvalue >1.0, (2) scree plot visualization, and (3) parallel analysis (1000 simulations). The one-factor component had an eigenvalue of 2.0 and explained 66.8% of the variance. The composite scale had adequate internal consistency (Cronbach α = 0.6708).

<u>Supplementary Table 3.</u> Principal components analysis of items measuring anticipated stigma for being uncircumcised (N=1503).

**Items**	**Mean (SD)**	**Factor Loading**	**Communality Estimate**
If your friends knew you are not yet circumcised, they would laugh at you.	2.55 (0.98)	0.8877	0.7880
If girls knew you are not yet circumcised, they would laugh at you.	2.63 (1.00)	0.8877	0.7880

Responses to each item included 1-strongly disagree, 2-disagree, 3-agree, to 4-strongly agree.
The unstandardized mean response to each item and its standard deviation (SD) are presented—data are only shown among complete cases. Following principal component analysis (PCA) using a polychoric correlation structure, several criteria were used to guide factor extraction following the PCA: (1) eigenvalue >1.0, (2) scree plot visualization, and (3) parallel analysis (1000 simulations). The one-factor component had an eigenvalue of 1.6 and explained 78.8% of the variance. The composite scale had adequate internal consistency (Cronbach α = 0.6666).

Supplementary Table 4. Sociodemographic characteristics of the study population stratified by health facility (N=1526).

	South Africa				Tanzania				Zimbabwe					
	**KMC** (n=98)	**BDH** (n=46)	**OFC** (n=158)	**EMC** (n=144)	**MRH** (n=255)	**MDH** (n=92)	**TMH** (n=132)	**MHC** (n=61)	**LBC** (n=49)	**BEC** (n=63)	**MPH** (n=143)	**NMC** (n=100)	**MDD** (n=147)	**SHC** (n=38)
**Age, y**														
10-14	50 (51.0)	29 (63.0)	115 (72.8)	82 (56.9)	189 (74.1)	79 (85.9)	124 (93.9)	49 (80.3)	16 (32.7)	33 (52.4)	68 (47.6)	54 (54.0)	66 (44.9)	13 (34.2)
15-19	48 (49.0)	17 (37.0)	43 (27.2)	62 (43.1)	66 (25.9)	13 (14.1)	8 (6.1)	12 (19.7)	33 (67.3)	30 (47.6)	75 (52.4)	46 (46.0)	81 (55.1)	25 (65.8)
**Primary Education**														
None/incomplete	12 (12.2)	24 (52.2)	88 (55.7)	64 (44.4)	191 (74.9)	74 (80.4)	123 (93.2)	57 (93.4)	30 (61.2)	20 (31.7)	135 (94.4)	85 (85.0)	122 (83.0)	24 (63.2)
Completed	86 (87.8)	22 (47.8)	70 (44.3)	78 (54.2)	64 (25.1)	18 (19.6)	9 (6.8)	4 (6.6)	19 (38.8)	43 (68.3)	8 (5.6)	15 (15.0)	25 (17.0)	14 (36.8)
**Religion**														
Christian	88 (89.8)	38 (82.6)	144 (91.1)	137 (95.1)	243 (95.3)	91 (98.9)	125 (94.7)	59 (96.7)	49 (100.0)	62 (98.4)	143 (100.0)	93 (93.0)	141 (95.9)	38 (100.0)
Muslim	0 (0.0)	1 (2.2)	1 (0.6)	0 (0.0)	5 (2.0)	1 (1.1)	6 (4.5)	2 (3.3)	0 (0.0)	0 (0.0)	0 (0.0)	2 (2.0)	3 (2.0)	0 (0.0)
Traditional	4 (4.1)	4 (8.7)	4 (2.5)	3 (2.1)	0 (0.0)	0 (0.0)	0 (0.0)	0 (0.0)	0 (0.0)	0 (0.0)	0 (0.0)	0 (0.0)	0 (0.0)	0 (0.0)
Agnostic/other	6 (6.1)	2 (4.3)	5 (3.2)	3 (2.1)	7 (2.7)	0 (0.0)	0 (0.0)	0 (0.0)	0 (0.0)	1 (1.6)	0 (0.0)	5 (5.0)	3 (2.0)	0 (0.0)
**Ever had sex** [a]														
No	72 (73.5)	38 (82.6)	135 (85.4)	105 (72.9)	204 (80.0)	77 (83.7)	123 (93.2)	40 (65.6)	32 (65.3)	52 (82.5)	126 (88.1)	86 (86.0)	139 (94.6)	30 (78.9)
Yes	25 (25.5)	7 (15.2)	22 (13.9)	38 (26.4)	51 (20.0)	15 (16.3)	9 (6.8)	21 (34.4)	17 (34.7)	11 (17.5)	17 (11.9)	14 (14.0)	8 (5.4)	8 (21.1)

Data are n (%). Proportions may not add up to 100% due to missing data.

[a] Refers to any sexual experience including mutual genital touching, oral, vaginal or anal sex.

See Figure 1 for study site names and study locations.

Supplementary Table 5. Perceived motivations to undergo VMMC stratified by age group and country.

Motivations to Undergo VMMC	South Africa 10-14 yrs (n = 276)	South Africa 15-19 yrs (n = 170)	Tanzania 10-14 yrs (n = 441)	Tanzania 15-19 yrs (n = 99)	Zimbabwe 10-14 yrs (n = 250)	Zimbabwe 15-19 yrs (n = 290)
Advice from others	39 (14.1)	13 (7.7)	216 (49.0)	26 (26.3)	24 (9.6)	16 (5.5)
School suggested it	9 (3.3)	6 (3.5)	47 (10.7)	3 (3.0)	3 (1.2)	0 (0.0)
Want to be healthy	14 (5.1)	17 (10.0)	44 (10.0)	20 (20.2)	24 (9.6)	32 (11.0)
To protect myself from HIV/STIs	184 (66.7)	134 (78.8)	196 (44.4)	83 (83.8)	189 (75.6)	252 (86.9)
To protect myself/partner from cancer	0 (0.0)	0 (0.0)	3 (0.7)	1 (1.0)	9 (3.6)	29 (10.0)
To improve hygiene/easier to clean	60 (21.7)	42 (24.7)	67 (15.2)	36 (36.4)	25 (10.0)	80 (27.6)
Make my penis more attractive	14 (5.1)	7 (4.1)	9 (2.0)	8 (8.1)	2 (0.8)	2 (0.7)
Heard sex will be better	2 (0.7)	6 (3.5)	2 (0.5)	5 (5.1)	0 (0.0)	9 (3.1)
Friends were doing/did it	19 (6.9)	7 (4.1)	69 (15.7)	24 (24.2)	7 (2.8)	12 (4.1)
To avoid stigma/shame/ridicule	1 (0.4)	0 (0.0)	17 (3.9)	7 (7.1)	0 (0.0)	3 (1.0)
To become a man	0 (0.0)	0 (0.0)	1 (0.2)	2 (2.0)	1 (0.4)	5 (1.7)

Data are n (%). Participants could provide multiple responses (unprompted).

Supplementary Table 6. Association of age group and perceived VMMC norms and anticipated stigma.

Characteristic	High Perceived Descriptive VMMC Norms			High Perceived Injunctive VMMC Norms			High Anticipated Stigma from Peers for Being Uncircumcised		
	% (n/N)	PR (95% CI)	aPR (95% CI)	% (n/N)	PR (95% CI)	aPR (95% CI)	% (n/N)	PR (95% CI)	aPR (95% CI)
**Age, years**									
10-14	49.0 (466/951)	**0.81 (0.72-0.90)**	**0.79 (0.71-0.89)**	46.7 (438/938)	0.87 (0.75-1.00)	0.86 (0.73-1.00)	41.6 (394/948)	**0.80 (0.71-0.91)**	**0.79 (0.68-0.90)**
15-19	53.9 (299/555)	Ref.	Ref.	49.1 (273/556)	Ref.	Ref.	41.3 (229/555)	Ref.	Ref.
**Country**									
South Africa	41.1 (175/426)	Ref.	Ref.	40.9 (177/433)	Ref.	Ref.	10.8 (47/434)	Ref.	Ref.
Tanzania	65.0 (351/540)	**1.57 (1.08-2.29)**	**1.59 (1.17-2.18)**	58.0 (302/521)	**1.46 (1.07-1.99)**	**1.49 (1.09-2.02)**	66.9 (354/529)	**5.59 (3.22-9.68)**	**5.60 (3.33-9.40)**
Zimbabwe	44.3 (239/540)	1.07 (0.64-1.79)	1.24 (0.85-1.82)	43.0 (232/540)	0.95 (0.57-1.57)	1.17 (0.71-1.93)	41.1 (222/540)	**2.65 (1.19-5.91)**	**3.05 (1.56-5.97)**
**Setting**									
Urban	42.0 (335/798)	Ref.	Ref.	39.1 (313/801)	Ref.	Ref.	39.0 (311/797)	Ref.	Ref.
Periurban	63.7 (158/248)	**1.55 (1.08-2.24)**	**1.49 (1.01-2.20)**	60.6 (149/246)	**1.73 (1.21-2.46)**	**1.62 (1.01-2.61)**	46.6 (115/247)	1.74 (0.70-4.37)	1.52 (0.89-2.60)
Rural	59.1 (272/460)	1.35 (0.84-2.18)	**1.46 (1.05-2.02)**	55.7 (249/447)	1.50 (0.96-2.33)	**1.58 (1.00-2.49)**	42.9 (197/459)	1.37 (0.57-3.31)	**1.89 (1.04-3.41)**

Prevalence ratios were estimated from modified Poisson regression models with generalized estimating equations and robust variance estimators. The multivariable models were complete-case analyses of 1506, 1494 and 1503 participants for perceived descriptive norms, perceived injunctive norms, and anticipated stigma, respectively.
Estimates in bold had a *P* value < 0.05.

Supplementary Table 7. Perceived level of HIV protection from VMMC stratified by age group and country.

Question	South Africa		Tanzania		Zimbabwe	
**Does circumcision protect a male from HIV?**	**10-14 yrs** (n = 273)	**15-19 yrs** (n = 170)	**10-14 yrs** (n = 441)	**15-19 yrs** (n = 99)	**10-14 yrs** (n = 250)	**15-19 yrs** (n = 290)
Yes, complete protection	55 (20.2)	34 (20.1)	69 (15.6)	9 (9.1)	23 (9.2)	6 (2.1)
Yes, some protection	105 (38.5)	85 (50.3)	193 (43.8)	68 (68.7)	163 (65.2)	252 (86.9)
No protection	50 (18.3)	39 (23.1)	55 (12.5)	11 (11.1)	12 (4.8)	18 (6.2)
Don't know	63 (23.1)	11 (6.5)	124 (28.1)	11 (11.1)	52 (20.8)	14 (4.8)
**Is a circumcised male's female sex partner protected from HIV?**	**10-14 yrs** (n = 271)	**15-19 yrs** (n = 167)	**10-14 yrs** (n = 441)	**15-19 yrs** (n = 99)	**10-14 yrs** (n = 250)	**15-19 yrs** (n = 290)
Yes, complete protection	38 (14.0)	8 (4.8)	33 (7.5)	14 (13.1)	8 (3.2)	4 (1.4)
Yes, some protection	82 (30.3)	93 (55.7)	49 (11.1)	27 (27.3)	118 (47.2)	186 (64.1)
No protection	54 (19.9)	45 (27.0)	159 (36.1)	32 (32.3)	47 (18.8)	53 (18.3)
Don't know	97 (35.8)	21 (12.6)	200 (45.4)	26 (26.3)	77 (30.8)	47 (16.2)

Data are n (%).

Supplementary Table 8. Perceived concerns about undergoing VMMC stratified by age group and country.

Perceived Concern	South Africa		Tanzania		Zimbabwe	
	**10-14 yrs** (n = 276)	**15-19 yrs** (n = 170)	**10-14 yrs** (n = 441)	**15-19 yrs** (n = 99)	**10-14 yrs** (n = 250)	**15-19 yrs** (n = 290)
Pain from procedure/injection	117 (42.4)	87 (51.2)	85 (19.3)	10 (10.1)	228 (91.2)	274 (94.5)
Duration of healing time	12 (4.4)	8 (4.7)	5 (1.1)	0 (0.0)	5 (2.0)	17 (5.9)
Sexual abstinence during wound healing	5 (1.8)	13 (7.6)	0 (0.0)	0 (0.0)	0 (0.0)	0 (0.0)
Potential damage to penis	9 (3.3)	10 (5.9)	0 (0.0)	0 (0.0)	2 (0.8)	3 (1.0)

Data are n (%). Participants could provide multiple responses (unprompted).
